# The integrated GOR-COVID-19 health monitor: quarterly reporting (“short-cycle monitoring”): Mare Derks

**DOI:** 10.1093/eurpub/ckac129.481

**Published:** 2022-10-25

**Authors:** M Derks, C Baliatsas, M Dückers, M Bosmans, E Marra

**Affiliations:** 1 Centre for Environmental Safety and Security, RIVM, Bilthoven, Netherlands; 2 Disasters and Environmental Hazards, NIVEL, Utrecht, Netherlands; 3 Impact, ARQ National Psychotrauma Centre, Diemen, Netherlands; 4 Faculty of Behavioural and Social Sciences, University of Groningen, Groningen, Netherlands

## Abstract

**Introduction/objective:**

Given the capricious nature of COVID-19, infections and accompanying restrictive measures to reduce spread of the virus are known to strongly vary over time. The impact of the corona crisis on the mental and physical health of the general population may fluctuate accordingly. The quarterly monitoring research line is aimed to monitor the health impact of the corona crisis over time on a high frequency basis.

**Methods:**

This is achieved through a multi-method approach, combining: a) survey data on perceived impact of corona measures and several health outcomes from two representative panels (∼ 5.000 adolescents/young adults aged 12 and 25 years; ∼ 5.000 adults aged 26+ years), and b) surveillance data of acute health problems from the Nivel Primary Care Database (∼1,6 million patients from 380 general practitioners). Data collection started in September 2021 and continues during the five-year monitoring program. Panel data is collected every quarter; Nivel surveillance data is collected weekly. Our contribution to the EUPHA conference will include data up and until September 2022 (i.e. five rounds of survey and surveillance data).

**Results:**

Preliminary results from September 2021 through March 2022 indicate that adolescents/young adults reported worse mental and physical health outcomes compared to adults. Moreover, they experienced a more negative impact of corona measures. Accordingly, their mental health related problems, including suicide ideation, spiked between December 2021 and February 2022, a period characterized by highly restrictive COVID-19-measures.

**Discussion:**

The quarterly monitoring research line showcase the relevance and feasibility of integrating multiple data sources in understanding the short- and long-term effect of the corona crisis. The results increase our understanding in the potential adverse effects of corona-related restrictive measures on population health, potentially aiding policy making and health promotion.

